# Automatic identification of anatomical landmarks in three-dimensional computed tomography/cone-beam computed tomography: a scoping review

**DOI:** 10.3389/fdmed.2026.1847046

**Published:** 2026-05-29

**Authors:** You Wu, Jing Zhai, Yufei Wang, Yawen Zhang, Xinghao Wang, Canlong Wang, Li Huang, Yan Jiang

**Affiliations:** 1 Department of Stomatology, The First Affiliated Hospital of Fujian Medical University, Fuzhou, China; 2 School of Stomatology, Fujian Medical University, Fuzhou, China; 3 School of Basic Medical Sciences, Fujian Medical University, Fuzhou, China; 4 Department of Stomatology, National Regional Medical Centre, Binhai Campus of the First Affiliated Hospital, Fujian Medical University, Fuzhou, China

**Keywords:** 3D computed tomography, artificial intelligence, automatic landmarking, deep learning, dentomaxillofacial deformities

## Abstract

**Objectives:**

This study aimed to conduct a scoping review to systematically review automatic identification techniques for soft-tissue and hard-tissue landmarks in three-dimensional (3D)-computed tomography (CT)/cone-beam computed tomography (CBCT), particularly focusing on artificial intelligence (AI)-based methods, to explore the progress and challenges in accuracy, efficiency, and clinical applicability.

**Materials and methods:**

In this scoping review, we searched for studies on automatic landmarking in CT/multi-slice spiral CT or CBCT that were published until January 2026 in PubMed, Web of Science, and the Cochrane Library. Studies that validated automatic landmarking methods against a reference standard, typically defined by manual annotations from human experts, were included.

**Results:**

In total, 37 (20 CBCT-only, 10 CT-only, and 7 CBCT + CT) studies were included in this review. Most of the studies focused on non-syndromic permanent dentition populations or a single type of malocclusion (except for 6 that involved mixed dentition) and had a limited number of samples, with <200 cases included in 75.7% of the studies. With 7–105 landmark annotations, most studies focused on hard-tissue marker points, and only 6 involved soft-tissue landmarks. Regarding accuracy, 56.8% of the studies met the clinical correctness standard [mean radial error (MRE) < 2 mm], and the overall trend indicated a gradual increase in the landmarking accuracy. Some studies did not use MRE or successful detection rate as an outcome measure, which potentially affected the overall comparability of the analysis.

**Conclusions:**

Existing studies have limited samples (number and type); the landmarks are mostly focused on hard tissues, and single algorithms are limited in their robustness and generalization performance in clinical applications. Furthermore, the system for evaluating the accuracy of 3D-automatic landmarking has not been standardized, and the clinical significance of traditional two-dimensional-accuracy thresholds in the 3D-space remains controversial.

## 1 Introduction

Dentofacial deformities refer to abnormal positional relationships among the teeth, maxilla, mandible, and facial soft tissues that affect masticatory function, temporomandibular joint health, and airway conditions. Conventional diagnosis relies on cephalometric analysis, whereby an orthodontist or maxillofacial surgeon manually marks anatomical landmarks to obtain clinically meaningful linear distance and angular data. Such manual calibration is time-consuming and dependent on experience, making the process prone to systematic errors. Considering these limitations, the rapid development of artificial intelligence (AI) has driven the automation of two-dimensional (2D) cephalometric measurements, primarily improving efficiency and stability (1–3). However, the shortcomings of 2D images, such as overlapping of the left and right sides of the skull, uneven magnification of structures (4), deformation of anatomical structures (5), and random errors owing to changes in head position when the patient is photographed, limit the accuracy and diagnostic comprehensiveness (5).

With the introduction of cone-beam computed tomography (CBCT) technology in the late 1990s, orthodontists rapidly adopted three-dimensional (3D) imaging, which provides significant advantages for specific diagnostic tasks and in indicated cases, can reduce the need for multiple 2D radiographs in orthodontic and maxillofacial surgical planning (6). Compared with computed tomography (CT)/multi-slice CT (MSCT), CBCT has a lower cost, less radiation exposure, and can directly measure the width of the maxilla and mandible from 3D images, thereby excluding the effects of tooth tilt or displacement. CBCT-based analysis is more accurate and constitutes the gold standard for maxillary and mandibular width analysis (7). Furthermore, CBCT can accurately assess key parameters, such as tooth position and airway structure, and quantify jaw-plane deviation, to provide an objective basis for surgical decision-making (8). Nevertheless, manual landmark identification in highly complex 3D images, such as those of the skull, remains a challenging and cumbersome task that is prone to random and systematic errors, which in turn affect the reproducibility of the assessment (6, 9). This limitation highlights the need to develop a high-precision AI-driven 3D-landmarking system, which when indicated, could improve the efficiency and accuracy of clinical diagnosis and treatment compared to manual methods.

In the development of automatic landmarking techniques, methods can be categorized into fully automatic and semi-automatic approaches based on the degree of manual intervention. Semi-automatic methods typically require human involvement, such as setting initial landmarks, adjusting parameters, or correcting inaccurate results; fully automatic methods rely on algorithms to independently complete the identification and localization of all landmarks. At present, many studies have been transitioning from semi-automatic to fully automatic approaches (2, 6); however, true fully automatic landmarking has not yet been achieved. Therefore, we believe that fully automated anatomical landmark recognition has not yet been achieved, and the term “automatic” used in this paper does not refer to true full automation.

Currently, the evolution of mainstream AI algorithms for automatic landmark localization follows a clear trajectory. In the early stages, forest models (10) and shape models (11, 12) were predominant, relying on handcrafted features and prior constraints. In later stages, deep neural networks became dominant, with convolutional neural networks (CNNs) (13–15) and U-Net architectures (16–20) facilitating end-to-end precise localization. Furthermore, graph convolutional networks (GCNs) (21, 22) have been used to model geometric relationships among landmarks, while multi-task learning (23, 24) and attention mechanisms (25) have further enhanced feature representation and localization accuracy. In terms of accuracy, mean errors of the included methods ranged from 0.89 ± 0.64 mm to 5.79 ± 0.98 mm, demonstrating a trend of continuous improvement alongside the advancement of neural networks.

Despite these technological advances, a comprehensive synthesis of the evidence on 3D automatic landmarking is lacking. Existing reviews often focus on 2D techniques, specific algorithms, or lack a systematic synthesis of methodological robustness, clinical applicability, and standardization across studies. Although the ideal perfect automatic landmarking would require no human intervention at all (truly fully automatic), in this review, “automatic landmarking” actually refers to methods that require only minimal user intervention, as truly fully autonomous systems have not yet been clinically validated. Therefore, this study employed a scoping review approach and systematically reviewed the techniques for automatic identification of hard-tissue and soft-tissue landmarks in 3D-CT/CBCT images, with a focus on AI-based analysis (e.g., deep learning), to explore the progress and challenges regarding accuracy, efficiency, and clinical applicability. The insights from this review can identify future research directions and inform the development of accurate, intelligent options for the diagnosis and treatment of craniofacial deformity.

## 2 Materials and methods

### 2.1 Study design and guidelines

This study was conducted strictly in accordance with the methodological framework of a scoping review, following the five-stage framework proposed by Arksey and O'Malley, and was reported in line with the PRISMA-ScR reporting guidelines.

### 2.2 Literature search strategies

To identify relevant articles, MeSH terms were combined with free-text phrases by using Boolean operators (“AND” and “OR”) to search the following databases: PubMed, Web of Science, and the Cochrane Library (for Cochrane systematic reviews) (last searched on 23 January 2026). As 3D-automated landmarking had not yet been mainstreamed with neural network technology by 2020, studies published up to and including 2020 were filtered using the keywords (“cephal*”) AND (“3D” OR “CBCT” OR “CT”) AND (“automated” OR “artificial intelligence” OR “machine learning” OR “deep learning” OR “learning”). For literature published between January 2021 and January 2026, wherein the application of neural networks in automated targeting had matured, and to avoid omitting literature on interdisciplinary research that involved whole-body (not skull-only) automated targeting, the following search terms were used: (“3D” or “CBCT” or “CT”) AND (“automated” OR “artificial intelligence” OR “machine learning” OR “deep learning” OR “learning” OR “AI-driven”). Duplicates were first eliminated using EndNote and then manually checked by one of the authors (JZ).

Studies included in this review were selected based on the following criteria, formulated according to the PICO (Population, Intervention, Comparison, Outcomes) framework:

(1) *Population:* Individuals undergoing CBCT or MSCT examination, without age restrictions.
(2) *Intervention:* Automatic anatomical landmark identification systems based on CBCT or MSCT images.
(3) *Comparison:* The performance of the automatic identification system was evaluated against a reference standard, typically consisting of manual annotations performed by human experts.
(4) *Outcomes:* The primary outcome measures were quantitative accuracy metrics for anatomical landmark identification (e.g., mean radial error, success detection rate). Studies that did not report quantitative accuracy outcomes were excluded.
(5) *Study design*: This study included *in vitro* and *in vivo* prospective and retrospective studies (clinical trials, comparative studies, validation studies, or evaluation studies) and excluded book chapters, animal studies, case reports, epidemiological studies, narrative reviews, and author's opinion articles.
(6) *Timeline setting*: No starting time restriction was set, and the search was conducted up to 23 January 2026. A phased search strategy was adopted for database retrieval. The first phase covered literature published in 2020 and earlier, while the second phase covered literature published between January 2021 and January 2026, with each phase employing a different set of search terms.
(7) *Language*: English.

### 2.3 Literature screening and data extraction

The literature screening and data extraction were conducted independently by two authors (YW and JZ).

**Figure 1:**
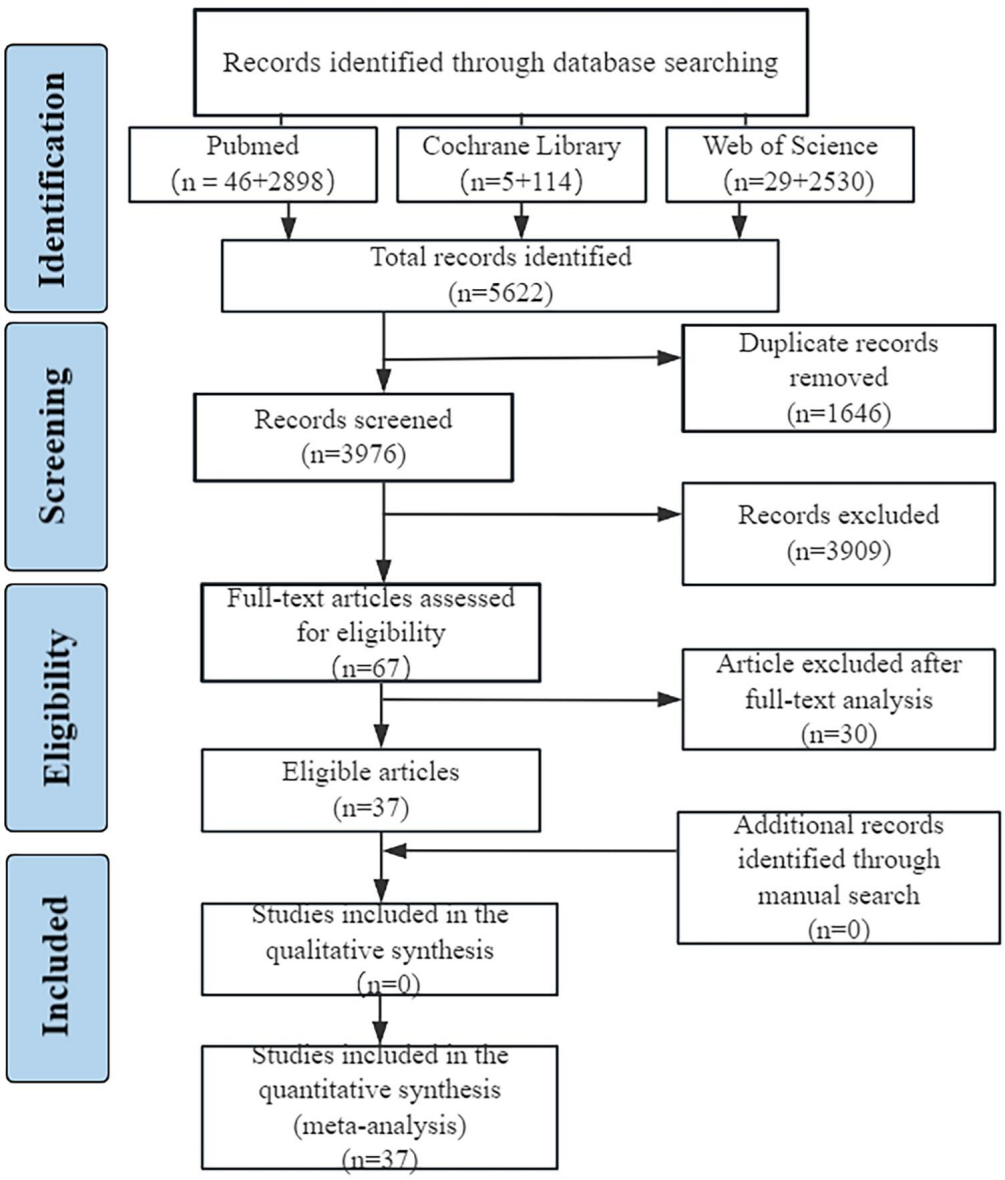
PRISMA flowchart nillustrating the study selection process. PRISMA, Preferred Reporting Items for Systematic Reviews and Meta-Analyses.

The search retrieved a total of 5,622 papers: 46 (through 2020) and 2,898 (2021–present) in PubMed; 5 (through 2020) and 114 (2021–present) in the Cochrane Library database; and 29 (through 2020) and 2,530 (2021–present) in the Web of Science database. After de-duplication, 3,976 papers were selected for preliminary screening. After title and abstract screening and full-text intensive reading, we excluded 3,909 publications that were not relevant to the automatic identification of anatomical landmarks in 3D-CT/CBCT or did not meet the research objectives. Moreover, two reviewers (YW and JZ) manually searched for references in original studies, reviews, and conference literature to supplement relevant studies that might have been missed by the electronic database search. Ultimately, 37 studies that met the inclusion criteria and provided valid data were included and analysed in depth. The literature inclusion screening process is detailed in a PRISMA flowchart diagram (Figure 1).

To assess information related to automatic landmarking in different studies, data were extracted on the datasets, sample sizes, general methods, algorithms, and accuracy evaluation metrics of the included literature.

### 2.4 Quality assessment

The methodological quality of the included diagnostic accuracy studies was assessed using the Quality Assessment of Diagnostic Accuracy Studies-2 (QUADAS-2) tool. This tool evaluates studies across four key domains: patient selection, index test, reference standard, and flow and timing. Each domain was assessed for risk of bias, and the first three domains were also assessed for concerns regarding applicability.

**Figure 2:**
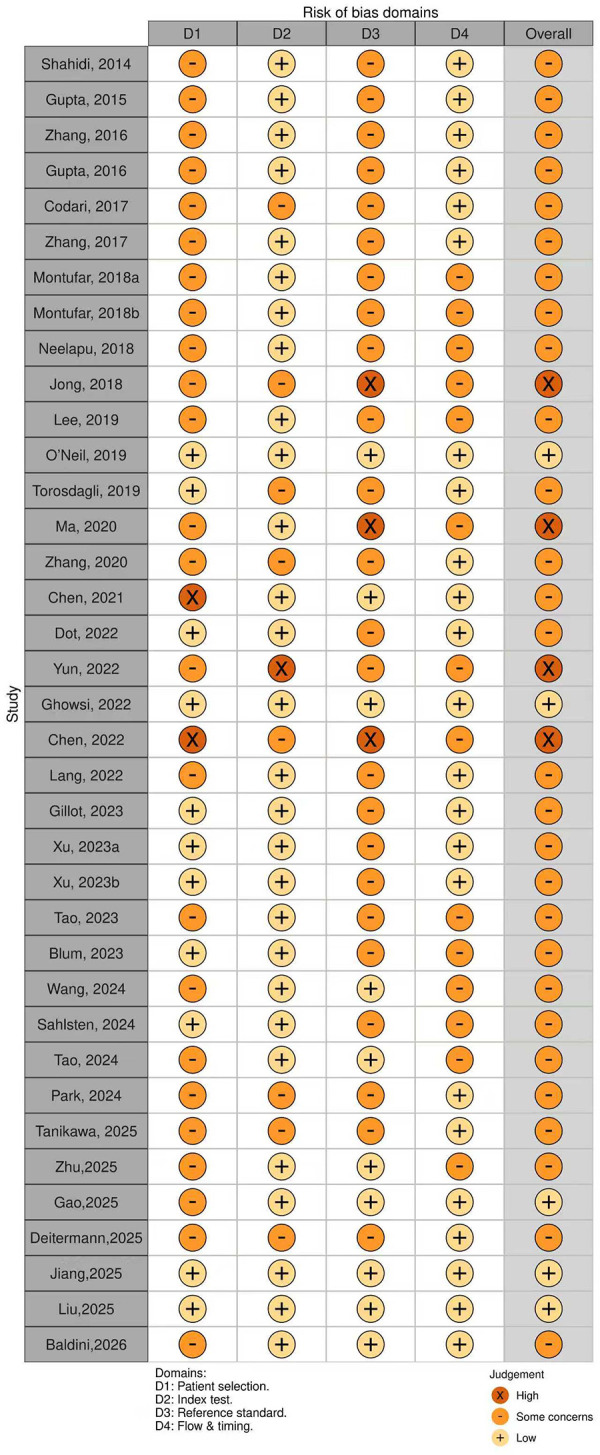
QUADAS-2: quality assessment of diagnostic accuracy studies.

Two reviewers (YW and JZ) independently performed the quality assessment for each included study. Any discrepancies in their judgments were resolved through discussion until a consensus was reached, or by consultation with a third senior reviewer if necessary. The results of this quality assessment are presented in Figure 2.

## 3 Results

### 3.1 Dataset characteristics

#### 3.1.1 Different types of 3D-Ct and types of samples (different stages of dentition and different types of malocclusion)

The reviewed studies comprised 20 CBCT-only cases, which primarily most of which involved the image analysis of patients without malocclusion (26–32), and 10 CT-only datasets, primarily used for surgical planning of various maxillofacial malformations (10, 13, 16, 21–24, 33–36). Most studies (Supplementary Table S1) included permanent dentition in their datasets, with only a few studies involving mixed dentition; only one study included cases of primary dentition. Furthermore, most studies focused on a certain type of patients (Supplementary Table S1), such as patients with cleft lip and cleft palate (21), patients with hemifacial atrophy (22), those with syndromic maxillofacial anomalies without syndromes (6, 13, 29–31), and patients who required orthognathic surgery for the treatment of dento-mandibular malformations (11, 16, 34–38) (Supplementary Table S1).

#### 3.1.2 Sample size and source

In the included studies, the sample sizes ranged from 18 to 1,190 cases, and 54.1% (20/37) had ≤ 100 (18–100) cases, and 21.6% (8/37) had 100–200 cases (107–198 cases). In recent years, an increasing number of studies have tended to use larger sample sizes (24.3%). For example, the study by Blum et al. included 1,045 cases (39), whereas the one by Liu et al. included 1,190 cases (17) (Supplementary Table S1).

Regarding the sample source, most data originated from a single medical center or were collected using uniform imaging equipment, and the participants were mostly limited to specific races or ethnic groups (e.g., adult Caucasian women (40), Korean adults (13, 24), and Japanese patients (41) (Supplementary Table S1).

### 3.2 Calibration of datasets

#### 3.2.1 Methods of calibration

The methods of manually calibrating 3D-CT landmarks are categorized into direct fixation on the 3D-reconstruction model and multiplanar reformation (MPR)-assisted fixation: 9 articles used the former and 14 articles used the latter. Datasets using direct fixation on the 3D-reconstruction model method (46–49) potentially had lower accuracy of automatic fixation than the MPR-assisted fixation methods (Supplementary Table S2).

#### 3.2.2 Types and numbers of landmarks

Most anatomical landmarks in previous 3D-CT automatic landmarking studies were limited to hard tissues, with only a few involving soft tissues (Supplementary Table S2), and most data were from patients with orthognathic surgery-related maxillofacial deformities (16, 36, 44). The number of landmarks ranged from 7 to 105, and most of them involved sagittal and vertical cephalometric indicators, with few indicators related to maxillary width discrepancy.

### 3.3 Technological innovations in automatic landmarking algorithms

#### 3.3.1 General method

The implementation of 3D-CT automatic landmarking can be divided into three different methods as follows:

(1) Knowledge-based methods: Based on predefined definitions, mathematical descriptions (such as peaks and nadirs) are applied to locate marker points on the anatomical contour of the image. In total, the studies in this review included four knowledge-based automatic point-fixation methods. For example, Gupta et al. detected 20 craniofacial landmarks by defining the volume of interest and contour features with an average error of 2.01 mm (27). The research team then implemented automatic head shadow measurement through geometric contour analysis and coordinate computation (28), with an accuracy close to that of manual marking. Neelapu et al. proposed an automatic algorithm for landmark detection on 3D-CBCT images based on the definition of human anatomical boundaries; its total average error at 20 landmarks was 1.88 mm (30). Montúfar et al. further innovatively combined a knowledge-based approach with a learning-based approach to propose a hybrid active-shaped model and a knowledge-based landmark localisation method (12). Such methods rely on prior knowledge but are less adaptable to complex anatomical structures.
(2) Atlas-based methods: Using an atlas of one or more reference images, the reference marker points are manually labelled, and then the reference image is aligned with the test image, whereby the reference marker points are transferred to the new image for landmarking. A total of 2 atlas-based automatic fixation methods were included in this review, e.g., Shahidi et al. combined feature alignment and atlas methods to achieve automatic identification of 14 landmarks with an average error of 3.4 mm (26). In another study, Codari et al. used a semi-automatic method to manually determine the mandibular nadir and, using intensity-based image alignment, transferred 21 landmarks from a reference skull to a specific skull to complete the automatic landmarking (40). The accuracy of this method is limited by image deformation and alignment errors.
(3) Learning-based methods: A training set is first used to train the AI to learn the image features, and thereby achieve automatic landmark localisation. This review included 32 learning-based automatic positioning methods, and their algorithms mainly included the forest model (10), shape model (11, 12), fully convolutional network (FCN) (33–35, 42), convolutional neural network (CNN) (13–15), graph convolutional network (21, 22), multi-task learning (23, 24), attention mechanisms (25), and U-Net Architecture (16–20). Among them, U-Net Architecture is frequently used with CNNs through cascade or parallel architectures (36, 37, 39, 43). The accuracy ranged from 0.89 ± 0.64 to 5.79 ± 0.98 mm, with a general trend of gradual improvement alongside neural network development, indicating greater accuracy than that of knowledge- and graph-based automatic fixation methods.

The automatic landmarking algorithms included in the literature are detailed in Supplementary Table S3.

### 3.4 Analysis of the accuracy of automatic landmarking

#### 3.4.1 Evaluation criteria for accuracy

The evaluation criteria for the accuracy of automatic landmarking—including mean absolute error (MAE), root mean square error (RMSE), concordance correlation coefficient (CCC), mean radial error (MRE), and successful detection rate (SDR)—vary in different studies. The MAE is the average of absolute errors between the predicted and actual values of all samples, and reflects the absolute size of the errors. RMSE is the square root of the average of the squares of errors between the predicted and actual values, which emphasises the contribution of larger errors. CCC measures the consistency between the predicted and actual values, which takes into consideration both relevance and bias. The SDR is the proportion of landmarks with measurement error less than a specific threshold. The MRE calculates the average Euclidean distance (usually in mm) between the automatically located points and manually annotated gold standard (Ground Truth, GT), where the Euclidean distance is the straight-line distance between two points in the N-dimensional space; the error distances of 2D and 3D landmarks originate from two and three coordinate directions, respectively. The more dimensions two points at the same spatial location exist in, the greater the error. The MRE and SDR are currently the most commonly used accuracy metrics (6, 10–18, 20–27, 29–32, 34–36, 38, 39, 41, 43, 45), with 30 of the 37 articles included in this review using this indicator (81.1%).

#### 3.4.2 Accuracy evaluation inconsistencies

The automatic landmarking accuracy of the available included literature is detailed in Supplementary Table S3 and ranged from 0.89 ± 0.64 mm to 5.79 ± 0.98 mm. Existing studies vary in the difficulty of manual calibration and automatic identification of the dataset; thus, it is not sufficiently objective to directly compare the accuracy of different studies by relying only on the MRE or SDR value of all points. For example, the overall mean error reported by Chen et al. was 1.64 ± 1.13 mm (25), which was smaller than the error in the results of Neelapu, et al. (30), at 1.88 ± 1.10 mm. However, the mean error for nasion in the study by Chen et al. was 1.69 ± 1.34 mm, which was larger than the 0.95 ± 0.69 mm mean error in the study by Neelapu et al.; for Point B, the values were 2.17 ± 1.64 and 1.78 ± 0.91 mm, respectively, and demonstrated a higher value in the study by Chen et al.

## 4 Discussion

CBCT is a medical imaging system that acquires 3D-volumetric data by a conical x-ray beam and a planar detector, which is characterised by sub-millimetre spatial resolution within a limited scanning range, with significant advantages of low radiation dose, low cost, and high spatial resolution (50). CT is a medical imaging technology that acquires cross-sectional images through the computerised reconstruction of data derived from the projection of x-ray beams around the human body from multiple angles. Compared to CBCT, CT has a wider scanning range, albeit with a relatively high radiation dose (51). As imaging parameters (e.g., voxel size, field of view, scanning time) of different devices may affect the accuracy of subsequent automatic landmarking (20), future studies should consider detailing the image-acquisition techniques and related parameters.

The tendency to include permanent dentition cases may be because permanent teeth have a relatively regular and stable anatomy, and thereby enable easy and accurate identification. Although accurate and reproducible identification of anatomical landmarks is not only key to the diagnosis and treatment of craniofacial anomalies (52), but also constitutes the foundation of AI-deep learning, adolescence is the optimal time for orthodontic treatment, because this developmental stage provides a critical window for improving facial profile and occlusal function (53) and poses greater challenges for automated landmark identification owing to greater anatomical variability during growth. Therefore, it is necessary to conduct targeted research. Notably, the focus on a certain type of patients in different studies limits the generalisability of their single algorithm. To enhance the clinical value and generalization of the algorithm, it is recommended that future studies should simultaneously include more diverse imaging data: different stages of dental development (deciduous, mixed, and permanent dentition periods), multiple types of malocclusion (Skeletal Classes I–III), and complex anatomical variants (e.g., cleft lip and palate, hemifacial atrophy, jaw lesions, and temporomandibular joint disorders).

The limitations of existing studies of 3D-CT automatic landmarking include the sample size and source, with 75.7% of the studies including fewer than 200 cases. Small-sample training is prone to model overfitting, which refers to good performance on a specific dataset but displays decreased accuracy when externally validated. A larger sample size effectively reduces the random error (54), which helps improve the accuracy of anatomical landmark localisation. Though a small number of studies have been conducted to increase the number of datasets for 3D-automatic localisation to more than 1,000 cases (39), the sample size of studies of 3D images has room for improvement compared to that of traditional 2D-image-based deep learning, which necessitates tens of thousands of samples for the training mode. Meanwhile, the homogeneity in sample composition of most existing studies tends to constrain the algorithm's ability to generalise across ethnicities. To overcome the impact of insufficient sample size and diversity on algorithm robustness and generalization, the study by Sahlsten et al. incorporated multicenter, multi-ethnic, and device-diverse data (both from Finland and Thailand) (38), and this model is expected to effectively support algorithm optimisation.

Regarding calibration methods, direct fixation on the 3D-reconstruction model is intuitive and conducive to the rapid identification of obvious body-surface landmarks (e.g., nasion, pogonion), but cannot completely avoid errors in the 3D-data-reconstruction process (55). In MPR-assisted fixation, sagittal, axial, and coronal slices can be displayed in a window around the 3D-rendering model. Multi-operator cross-validation through multi-view assessment can reduce interobserver variability and enhance the accuracy of landmark identification (9). However, Hassan, et al. (9) showed that, when using the MPR method, the average time for landmark calibration doubled, though the accuracy improved. This precision advantage trend that was observed in manual landmark identification with MPR assistance may extend to deep learning-based automated landmark-detection algorithms. Despite excluding the potential effect of sample-size differences, in the studies by de Jong, et al. (29) and Montúfar et al. (11), the MRE of the nasion in MPR-assisted landmarking decreased by 0.65 mm compared to that of direct fixation on 3D-reconstructed models for landmarking. As human experts show higher accuracy when using MPR-assisted landmarking, smarter algorithms can be developed by allowing AI to systematically learn from the aforementioned high-quality dataset.

Most anatomical landmarks in previous 3D-CT automatic landmarking studies mainly focused on hard tissues, and relatively few studies investigated the identification of soft-tissue marker points (52). This phenomenon may stem from two factors: on the one hand, CT/CBCT imaging techniques have higher contrast resolution for hard tissues; on the other hand, many algorithms actively exclude soft-tissue information in the pre-processing stage, which may introduce noise and affect the detection accuracy (14). In addition, compared with the identification of bony anatomical landmarks, the automatic identification of soft tissue anatomical landmarks faces more prominent challenges, representing an important reason for the data gap in this field. First, soft tissues exhibit low contrast and blurred boundaries in commonly used imaging modalities (4), and their morphology is susceptible to non-anatomical factors such as patient positioning (20) and physiological status (4), increasing the difficulty of model learning. Second, clinical definitions of soft tissue landmarks often lack a unified anatomical consensus, and inter-expert annotation consistency is low, resulting in considerable observer variability in the “gold standard” itself (47, 49) and consequently affecting the reliability of model training supervision and accuracy evaluation. Furthermore, the insufficient availability of soft tissue samples in public datasets limits the training efficacy and generalization capability of deep neural networks for such tasks (20). Although the accurate identification of soft tissues relies more on techniques such as three-dimensional morphologic distance mapping, with the continuous advancement of CBCT imaging, some researchers have recently started applying CBCT to facial soft-tissue studies (56, 57), which provides new possibilities. Considering the region-specific variations in the accuracy of CBCT-based measurement of soft tissues in the study by Choudhary, et al. (56), future studies should consider improving the soft-tissue landmarking algorithm of CBCT to enhance its comprehensiveness in the measurement and analysis of facial 3D morphology. Such improvement can expand the application value of CBCT in clinical scenarios, such as orthodontic treatment design and plastic surgery planning.

With rapidly developing AI technology, data-driven computer vision neural network architectures (CNN, FCN, and U-Net Architectures) have become research hotspots in the field of learning-based automatic fixation owing to their powerful feature-extraction capabilities. In early machine learning, Zhang et al. proposed a segment-guided partial joint regression forest model to automatically digitise craniofacial landmarks on CBCT with regression forests and multiscale statistical features, with an average error of 1.44 mm in calibrating 15 landmarks (10); in 2018, Montúfar et al. used Active Shape Model to locate the landmarks on orthogonal projection and then convert them to 3D coordinates, with an average error of 3.6476 mm in calibrating 18 landmarks (11). Next, they proposed a Hybrid Active Shape Model with Knowledge-based Landmark Localization (12), which further reduced the average error to 2.5129 ± 1.6058 mm. Subsequently, Zhang et al. proposed a context-guided FCN for joint bone segmentation and landmark digitisation, with an average error of only 1.10 mm for 15 landmarks (33). Since 2019, neural networks, including CNNs, FCNs, and U-Net Architectures, have received large-scale attention, and their accuracies have been gradually improved. For example, O’Neil et al. proposed the use of FCN (42), instead of the traditional decision forest, for automatic localisation of anatomical landmarks in CT scans, whereby FCN performed significantly better than the decision forest method. Lee et al. used a machine-learning method of shaded 2D images for automated 3D-cephalometric annotation, using multiple 2D-shaded images with 3D-geometric morphology information, combined with VGG-net for training, with an average error of 1.5 mm for 7 main landmarks (13). These neural network-based methods learn the features of the landmarks through a large amount of training data and can effectively adapt to the different craniomaxillofacial differences of individuals and improve the accuracy of automatic landmarking. However, the challenges of such computer vision-based algorithmic model training include reliance on large-scale and high-quality labelled data, high demand for computational resources during training, and weak model interpretability. Current research trends in this field include methods to further enhance model robustness by incorporating attention mechanisms (18, 25) and multi-task learning (23, 24). Chen et al. employed a graph attention module and a self-attention gating module (25), where the graph attention module is used to capture global-local dependencies between landmarks, whereas the self-attention gating module enhances the focus on key information, and thereby improves the accuracy of landmark detection. Overall, the deep-learning based automatic landmarking method is more accurate than methods based on knowledge and graphs and other machine-learning algorithms.

Regarding accuracy, the relatively low localisation accuracy reported in some literature may be attributed to the complexity of the sample type, which makes the same anatomical structures more difficult to identify owing to age, trauma, and developmental deformities, and increases the overall mean error. The study by Park et al. further confirmed that in young permanent teeth with incomplete root development, the flared opening feature of the apical foramen that had not yet fully closed resulted in a significant reduction in the 3D-spatial localisation accuracy of the anatomical apical landmarks (58). Therefore, when assessing the accuracy of algorithms, the overall average MRE or SDR cannot be used as the only comparative metric for the following reasons. First, the clinical acceptability of an MRE < 2 mm is context-dependent. In 2D analysis, this threshold may align with routine diagnostic requirements. However, in 3D CBCT, an MRE < 2 mm alone is insufficient, because angular measurements are highly sensitive to directional error accumulation, particularly in anatomically indistinct regions. Thus, MRE should be assessed alongside spatial measurement parameters rather than serving as the sole criterion for clinical applicability (49). Second, the SDR should be defined as the proportion of landmarks with radial errors within thresholds of 2 mm, 3 mm, and 4 mm (59). Third, the clinical impact of a 2-mm error in a single landmark depends on its role in subsequent measurements. For core landmarks (e.g., sella and nasion), such errors can propagate through angular and linear measurements (e.g., ANB angle), potentially biasing diagnostic interpretation, particularly when values lie near clinical decision thresholds (49). Finally, human experts are not a perfect gold standard. Inter-/intra-observer variability and anatomical ambiguity (e.g., gonion, the root apex of flared canals) introduce inherent limitations to manual annotations (49). Thus, even after AI-based landmarking, manual secondary calibration is recommended to ensure clinical reliability.

The existing accuracy evaluation system has some limitations. The MRE and SDR, as the core indices for evaluating the localisation performance of AI models, can effectively reflect the Euclidean distance between the algorithm output points and GT points, as well as facilitate stability detection. However, in clinical applications, these indicators have some limitations and need to be considered in conjunction with anatomical characteristics and clinical needs. For example, the bilateral Porion (Po) lacks clear bony structural transitional characteristics. It is especially difficult to accurately locate by imaging features in the *X*-axis and *Y*-axis directions; however, as long as the Z-axis (vertical direction) positioning is accurate, and based on the Po points to establish the Frankfort Horizontal Plane, the functional needs of clinical orthodontic analysis or surgical planning can still be met (60). While MRE is used in many studies, others do not use it, affecting the overall comparability of the analysis. To enhance comparability across future studies, we propose standardizing the evaluation of automated 3D cephalometry by translating landmark accuracy into clinically interpretable parameters (e.g., angles, distances, and areas) with predefined thresholds (≤ 2.0 mm/°), rather than relying solely on MRE (61).

Some scholars have transformed the point coordinates output from automatic landmarking into measurements in clinical diagnosis as a complementary method to assess the accuracy of the landmarking method (28, 35, 44). As head shadow measurements in the median sagittal plane of CBCT do not significantly differ from those of traditional 2D measurements (62), some studies validated algorithmic accuracy by comparing orthogonal projection measurements (e.g., line distances and angles) in the median sagittal plane of 3D images by AI and GT (35), or compared the knowledge-based automated landmarking method in Angle Class I patients with manual landmarking of the 3D-line distance and angle obtained from measurements (28). Other studies assessed the performance of the algorithms by comparing the differences between GT and AI in 53 measures (44). However, these methods could not fully utilise the potential of 3D measurements, especially for eccentric patients, where the projected angle in the median sagittal plane may significantly deviate from the true 3D angle owing to mandibular deviation. Therefore, this study propose the introduction of a potential complementary evaluation method: comprehensive 3D-measurements of landmark coordinates acquired by deep learning and their statistical comparison with manual landmarking measurements generated by human experts to validate their accuracy after automated landmarking to comprehensively assess the clinical applicability of the AI model in the 3D-space.

To address the current limitations in the field, future research should focus on the following directions. First, larger and more diverse datasets should be constructed, covering multiple imaging modalities, anatomical sites, and population characteristics, while improving the standardized annotation of soft tissue landmarks. Second, self-supervised or semi-supervised learning frameworks should be developed to reduce reliance on large-scale annotated data, combined with the integration of anatomical prior knowledge (e.g., geometric constraints introduced by graph neural networks) to enhance model generalization across different clinical scenarios. Third, standardized evaluation systems should be established, including unified evaluation metrics, fixed data splits, and public benchmarking platforms, to facilitate fair comparisons between studies and accelerate the clinical translation of automatic landmarking techniques.

### 4.1 Limitations

Several methodological limitations of this review may affect the comprehensiveness of the findings and need to be addressed in future research: (1) the potential omission of grey literature (e.g., conference papers, technical reports); (2) the significant heterogeneity across included studies in terms of datasets, algorithms, and evaluation metrics, which limits direct comparisons between studies; (3) the inability to perform a meta-analysis due to this variability; (4) the potential bias introduced by single-center, small-sample datasets, which limits the generalizability of the results. Future studies should adopt multicenter designs, incorporate larger and more diverse datasets, and employ standardized evaluation frameworks.

## 5 Conclusions

In recent years, with the continuous advancement of algorithms, 3D-CT automatic landmarking technology has significantly advanced. However, the current studies still face the following key challenges: (1) limited algorithm robustness due to small, homogeneous datasets often focused on non-syndromic permanent dentition population or single malocclusion types; (2) limited number of landmarks and constrained selection, with few studies focusing on soft-tissue landmarks, thus limiting the algorithm's generalization in clinical applications; (3) a non-standardized 3D-accuracy evaluation system, with most studies following the traditional thresholds of 2D-cephalometric measurements (e.g., A Euclidean distance < 2 mm is considered the criterion of clinical accuracy and distances < 4 mm are considered clinically acceptable); and (4) the lack of systematic studies that validate the effects of dimensional differences in Euclidean distance on angular measurement data and its clinical significance, as well as unclear evaluation criteria that are more closely correlated with clinical significance. Future studies need to address these issues to further the development of 3D-CT auto-pointing technology and its better application in clinical practice.
