# Supplementary material for: Automatic identification of anatomical landmarks in three-dimensional computed tomography/cone-beam computed tomography: a scoping review
Source: Front Dent Med. 2026 May 29;7:1847046. doi: 10.3389/fdmed.2026.1847046 (PMC13260512; doi:10.3389/fdmed.2026.1847046)
Supplement: Supplementary file 1 [file Table1.docx]

**Table 1.** Composition and features of the datasets from the included studies

| **Year, author** | **Quantity of CBCT/CT** | **Imaging Device; CBCT/CT parameters** | **Age (y)/dentition type; data source, presence and classification of malocclusion** |
| --- | --- | --- | --- |
| 2014, Shahidi et al.^26^ | 28/0 | NewTom VGi Cone Beam CT machine (QR SRL Company, Verona, Italy);  tube current: 4.71 mA, tube voltage: 110 kVp, scan time: 3.6 s, FOV: 15×15 cm | 10–43/MD;  Orthodontic patients without significant fractures or severe skeletal anomalies |
| 2015, Gupta et al.^27^ | 30/0 | iCAT Next Generation CBCT unit (Imaging Sciences International, Hatfield, PA);  scan time: 26 s, FOV: 17×22 cm, isometric voxel size: 0.25–0.40 mm | NM/NM;  Datasets were collected randomly from the postgraduate orthodontic clinic database, irrespective of age, sex, and ethnicity. |
| 2016, Zhang et al.^10^ | 41/30 | Machine: NM;  CBCT’s voxel size: 0.4 mm;  MSCT’s voxel size: 0.488×0.488×1.25 mm³ | NM/NM;  Patients with non-syndromic dentofacial deformity (Class II, Class III jaw deformity), additional training dataset (Class I) |

**Table 1 (continued)**

| **Year, author** | **Quantity of CBCT/CT** | **Imaging Device; CBCT/CT parameters** | **Age (y)/dentition type; data source, presence and classification of malocclusion** |
| --- | --- | --- | --- |
| 2016, Gupta et al.^28^ | 30/0 | iCAT next generation CBCT unit (Imaging Sciences International, Hatfield, PA);  isometric voxel size: 0.25–0.40 mm | NM/NM;  All CBCT images were collected randomly from the archives of orthodontic treatment clinic database irrespective of age, gender, and ethnicity |
| 2017, Codari et al.^40^ | 18/0 | Machine: NM;  tube current: 6–10 mA (pulse mode), tube voltage: 105 kV, FOV: 20×17 cm, X-ray generator: fixed anode | 37–74/PD;  Adult healthy Caucasian women |
| 2017, Zhang et al.^33^ | 77/30 | Machine: NM;  CBCT’s voxel size: 0.3 or 0.4 mm; MSCT’s voxel size: 0.488×0.488×1.25 mm³ | NM/NM;  Non-syndromic dentofacial deformities patients (Skeletal Class I, Skeletal Class II, Skeletal Class III) |

**Table 1 (continued)**

| **Year, author** | **Quantity of CBCT/CT** | **Imaging Device; CBCT/CT parameters** | **Age (y)/dentition type; data source, presence and classification of malocclusion** |
| --- | --- | --- | --- |
| 2018, Montúfar et al.^11^ | 30/0 | Machine: NM;  Parameter: isometric voxel: 0.4 mm | NM/NM;  The CBCT images were randomly selected from a public data set: the Virtual Skeleton Database from the Swiss Institute for Computer Assis ted Surgery Medical Image Repository |
| 2018, Montúfar et al.^12^ | 24/0 | Machine: NM;  Parameter: NM | NM/NM;  CBCT head volume scans were from the Virtual Skeleton Database from the Medical Image Repository of the Swiss Institute for Computer Assisted Surgery |

**Table 1 (continued)**

| **Year, author** | **Quantity of CBCT/CT** | **Imaging Device; CBCT/CT parameters** | **Age (y)/dentition type; data source, presence and classification of malocclusion** |
| --- | --- | --- | --- |
| 2018, Neelapu et al.^30^ | 30/0 | iCAT Next Generation CBCT unit (Imaging Sciences International, Hatfield, PA);  scan time: 26 s, FOV: 17×23 cm, isometric voxel size: 0.25–0.40 mm | NM/NM;  Data sets were collected retrospectively from the archived database of the post-graduate orthodontic clinical database irrespective of age, gender and ethnicity |
| 2018, Jong et al.^29^ | 39/0 | Machine: NM;  FOV: from the mandible to at least 1 cm  above the orbits (generally including the facial skeleton), slice thickness: 0.3–1 mm | 16–54/PD;  A non-syndromic cohort from the Oral and Maxillofacial Surgery and Special Dental Care department at Erasmus MC, Rotterdam, The Netherlands |
| 2019, Lee et al.^13^ | 0/27 | Machine: NM;  Parameter: NM | Mean age=24.22±2.91/PD;  Normal Korean adults with skeletal class I occlusion |

**Table 1 (continued)**

| **Year, author** | **Quantity of CBCT/CT** | **Imaging Device; CBCT/CT parameters** | **Age (y)/dentition type; data source, presence and classification of malocclusion** |
| --- | --- | --- | --- |
| 2019, O’Neil et al.^42^ | 0/20 | Canon, Siemens, G.E., Philips;  Parameter: NM | NM/NM;  Retrospective random selection of previous cranial CT scan samples (including haemorrhage, tumours and age-related change, pathological manifestations, and basically balanced gender) |
| 2019, Torosdagli et al.^37^ | 50/0 | CB MercuRay CBCT system (Hitachi Medical Corporation, Tokyo, Japan);  tube current: 10 mA, tube voltage: 100 kVp, FOV: 12 inch (about 30.5 cm) | Mean age=22.4±9.6/PD;  Patients with various craniofacial deformities (including congenital, developmental and anatomical variations) |

**Table 1 (continued)**

| **Year, author** | **Quantity of CBCT/CT** | **Imaging Device; CBCT/CT parameters** | **Age (y)/dentition type; data source, presence and classification of malocclusion** |
| --- | --- | --- | --- |
| 2020, Ma et al.^14^ | 0/66 | TOSHIBA CT (Aquilion ONE ViSION, Toshiba Medical System Corp. Tochigi 324-8550, Japan);  pixel size: 0.351 mm, width and height: 512×512 | NM/NM;  An OMS patient database during 2008–2017 at The University of Tokyo Hospital was evaluated to select data |
| 2020, Zhang et al.^34^ | 77/30 | Machine: NM;  CBCT’s voxel size: 0.30 or 0.40 mm,  MSCT’s voxel size: 0.488×0.488×1.25 mm³ | NM/PD;  Patients with non-syndromic dentofacial deformities (Skeletal Class I、Skeletal Class II、Skeletal Class III) |
| 2021, Chen et al.^43^ | 80/0 | Machine: NM;  voxel size: 0.30–0.40 mm | NM/NM;  Patients with CMF deformities |

**Table 1 (continued)**

| **Year, author** | **Quantity of CBCT/CT** | **Imaging Device; CBCT/CT parameters** | **Age (y)/dentition type; data source, presence and classification of malocclusion** |
| --- | --- | --- | --- |
| 2022, Dot et al.^35^ | 0/198 | Machine: NM;  average slices: 744, mean in-plane pixel size: 0.45×0.45 mm^2^, mean FOV: 22.9 cm, mean slice thickness: 0.33 mm | 14–60, mean age=27±11/PD;  Presurgical CT scans from ethnically diverse patients with various dentofacial deformities eligible for combined orthodontic-surgical treatment |
| 2022, Yun et al.^24^ | 0/24 | Machine: NM;  Parameter: NM | Mean age=24.22±2.91/PD (adults);  24 calibrated subjects with normal skeletal class I occlusion and 229 anonymized subjects with dentofacial deformities and malocclusions |

**Table 1 (continued)**

| **Year, author** | **Quantity of CBCT/CT** | **Imaging Device; CBCT/CT parameters** | **Age (y)/dentition type; data source, presence and classification of malocclusion** |
| --- | --- | --- | --- |
| 2022, Ghowsi et al.^6^ | 100/0 | Imaging Science International CBCT scanner (Hatfield, PA) (95%), NewTom device (Verona, Italy) (5%);  FOV: ≥16×13 cm, voxel size: 0.3 mm | NM/PD;  Exclusion criteria: restorative work with significant scatter and presence of craniofacial deformities, syndromes, or cleft lip and palate |
| 2022, Chen et al.^25^ | 89/33 | Machine: NM;  after standardization: isotropic voxel spacing: 1 mm, average resolution: 518×518×384 | NM/NM;  PDDCA dataset (a Public Domain Database for Computational Anatomy) contains 48 CT images from the Radiation Therapy Oncology Group (RTOG) 0522 study |
| 2022, Lang et al.^23^ | 50/45 | Machine: NM;  CBCT: 0.4 mm or 0.3 mm (>536×536×440);  spiral CT: 0.49×0.49×1.25 mm³ | 37–80/PD;  Patients with non-syndromic jaw deformities, where 20 patients are with CMF defects |

**Table 1 (continued)**

| **Year, author** | **Quantity of CBCT/CT** | **Imaging Device; CBCT/CT parameters** | **Age (y)/dentition type; data source, presence and classification of malocclusion** |
| --- | --- | --- | --- |
| 2023, Gillot et al.^31^ | 143/0 | Machine: NM;  77 cases: 0.3–0.4 mm,  66 cases: 0.08–0.16 mm | NM/NM;  Patients without craniofacial anomalies or syndromes and scans with artefacts produced by orthodontic appliances |
| 2023, Xu et al.^22^ | 0/135 | Machine: NM;  Parameter: NM | 7–29, mean age=13±5/MD+PD;  All patients had a diagnosis of nonsyndromic HFM. Patients without bone defects, a history of craniomaxillofacial surgery, or genetic diseases were excluded |
| 2023, Xu et al.^21^ | 0/117 | Machine: NM;  Parameter: NM | 11–31, mean age=16±5/MD+PD;  Patients with maxillary retrusion and facial asymmetry |

**Table 1 (continued)**

| **Year, author** | **Quantity of CBCT/CT** | **Imaging Device; CBCT/CT parameters** | **Age (y)/dentition type; data source, presence and classification of malocclusion** |
| --- | --- | --- | --- |
| 2023, Tao et al.^16^ | 0/80 | Machine: NM;  pixel size: 0.45 mm×0.45 mm, slice interval: 1 mm, resolution: 512×512×231 | NM/NM;  Patients with dentomaxillofacial deformities requiring orthognathic-orthodontic joint treatment, excluding cases with congenital dentofacial deformities or those with prior orthognathic treatment |
| 2023, Blum et al.^39^ | 1045/0 | Galileos® Comfort Plus, Dentsply Sirona (Bensheim, Germany);  tube current: 5 mA, tube voltage: 98 kV, radiation time: 14 s, axial slice thicknesses: 0.287 mm and 0.250 mm, isotropic voxels: 512×512×512 and 616×616×616 | Mean age=37.1±19.7/PD;  All CBCT datasets were acquired between 2013 and 2020 in the Department of Oral and Maxillofacial Surgery |

**Table 1 (continued)**

| **Year, author** | **Quantity of CBCT/CT** | **Imaging Device; CBCT/CT parameters** | **Age (y)/dentition type; data source, presence and classification of malocclusion** |
| --- | --- | --- | --- |
| 2024, Wang et al.^32^ | 450/0 | Meyer software (mDX-13STSP1A, Hefei, Anhui);  tube current: 5 mA, tube voltage: 120 kV, exposure time: 20 s, FOV: 23×18 cm, nominal focal point: 0.5×0.5 mm | 18–45, mean age=28.02±8.03/PD;  Eligible cases had 0° < ANB angle < 4°, normal overjet and overbite and no missing teeth except for the third molars, excluding those with growth disorders, craniofacial anomalies (cleft/syndromes/trauma/tumour), or prior facial surgeries |
| 2024, Sahlsten et al.^38^ | 309/0 | DentiScan (NSTDA), Viso G7 (Planmeca), Scanora 3Dx (Soredex);  voxel size: 0.15 mm, 0.2 mm, 0.25 mm, 0.3 mm, 0.4 mm, 0.45 mm | NM/NM;  Patients undergoing orthognathic or facial surgery, including patients with normal anatomy and anatomical deformities |

**Table 1 (continued)**

| **Year, author** | **Quantity of CBCT/CT** | **Imaging Device; CBCT/CT parameters** | **Age (y)/dentition type; data source, presence and classification of malocclusion** |
| --- | --- | --- | --- |
| 2024, Tao et al.^36^ | 0/74 | Machine: NM;  resolution: 512×512×231, pixel size: 0.45 mm×0.45 mm, slice interval: 1 mm | Mean age=23.3/PD;  Patients with dentomaxillofacial deformities requiring orthognathic-orthodontic joint treatment, excluding congenital cases or those with prior orthognathic surgery |
| 2024, Park et al.^44^ | 80/0 | i-CAT 17-19 device (Imaging Sciences International, Hatfield, PA, USA);  Parameter: NM | NM/NM;  NM |

**Table 1 (continued)**

| **Year, author** | **Quantity of CBCT/CT** | **Imaging Device; CBCT/CT parameters** | **Age (y)/dentition type; data source, presence and classification of malocclusion** |
| --- | --- | --- | --- |
| 2025, Tanikawa et al.^41^ | 185/0 | Alphard-3030 (Asahi Roentgen Ind. Co. Ltd., Kyoto, Japan);  tube current: 2 mA, tube voltage: 80 kV, FOV: 20×20 cm, voxel size: 0.39 mm | 18–35/PD;  Exclusion criteria: a history of trauma or injury to the face, congenital anomalies, significant abnormal bone defects (such as tumours), significant artefacts in the CBCT data, and absence of more than three teeth (except third molars) |

**Table 1 (continued)**

| **Year, author** | **Quantity of CBCT/CT** | **Imaging Device; CBCT/CT parameters** | **Age (y)/dentition type; data source, presence and classification of malocclusion** |
| --- | --- | --- | --- |
| 2025, Zhu et al.^18^ | 287/200 | CBCT: Morita 3-D Accuitomo scanner (J. Morita, Japan);  tube current: 5 mA, tube voltage: 85 kV, scan time: 17.5 s, voxel size: 0.25 mm, resolutions ranging from 256×256×141 to 565×405×256 (average: 561×403×143);  CT: Philips MX 16-slice scanner (Philips, Dongqin);  tube current: 230 mA, tube voltage: 120 kV, slice thickness: 0.5 mm, interslice spacing: 0.5 mm, resolutions: from 256×256×196 to 512×666×279 | CBCT: 16–60, mean age=33.2±7.09/PD; Data were collected from March 2022 to October 2022;  CT: 16–60, mean age=29.7±8.41/PD;  Data were collected from the imaging database of the West China School/Hospital of Stomatology, Sichuan University, covering patients from September 2020 to March 2022 |
| 2025, Gao et al.^45^ | >420/0 | CBCT volumes: NewTom VGi evo (Cefla, Imola, Italy) ;  tube current: 3–8 mA, tube voltage: 110 kV, FOV: 15×15 cm, voxel size: 0.2–0.3 mm | CephNet: 12–45, mean age=18.7/PD; DentalFusion: 16–38, mean age=19.3/PD; Three datasets compiled from clinical archives at multiple orthodontic centres |

**Table 1 (continued)**

| **Year, author** | **Quantity of CBCT/CT** | **Imaging Device; CBCT/CT parameters** | **Age (y)/dentition type; data source, presence and classification of malocclusion** |
| --- | --- | --- | --- |
| 2025, Deitermann et al.^19^ | 0/287 | SOMATOM Definition Flash and SOMATOM Definition AS from Siemens (Erlangen, Germany);  FOV: from 195×195×146.3 mm to 439×439×600 mm; voxel sizes: from 0.38×0.38×0.7 mm to 0.86×0.86×4.0 mm | Data from RWTH Aachen University Hospital; Patients under 20 and with skeletal mandibular diseases were excluded |

**Table 1 (continued)**

| **Year, author** | **Quantity of CBCT/CT** | **Imaging Device; CBCT/CT parameters** | **Age (y)/dentition type; data source, presence and classification of malocclusion** |
| --- | --- | --- | --- |
| 2025, Jiang et al.^20^ | 498/0 | i-CAT next generation machine (Imaging Sciences International, Hatfield, PA, USA);  scan time: 26 s, FOV: 17×22 cm, isometric voxel size: 0.25–0.40 mm | 4–46, mean age=17.64±5.44/DD+MD+PD;  We retrospectively collected CBCTs from the First Affiliated Hospital of Fujian Medical University over the past 7 years；  Including: (1) CBCT scans with voxel size of 0.25–0.4 mm3 and FOV ≥17 × 22 cm; (2) patients of all ages, sexes, and types of malocclusions and skeletal patterns. Excluding: (1) imaging processes exhibiting significant scatter during restorative work; (2) patients with incomplete clinical data or who declined to participate |

**Table 1 (continued)**

| **Year, author** | **Quantity of CBCT/CT** | **Imaging Device; CBCT/CT parameters** | **Age (y)/dentition type; data source, presence and classification of malocclusion** |
| --- | --- | --- | --- |
| 2025, Liu et al.^17^ | 390/800 | Philips MX 16 CT, Philips Brilliance 64 GE Discovery CT750;  Spiral CT: slice interval: 0.32–1.0 mm, thickness: 0.625–1.0 mm, pixel size: 0.45mm (0.32–0.63 mm), Matrix: 5123;  CBCT: tube current: 3–14 mA, tube voltage: 70–120 kV, voxel size: 0.25 mm (0.15–0.40 mm), Matrix: 610–670^3^ | 6.5–69.5, mean age=30.41±8.65/MD+PD;  Including patients with malocclusion, missing dental landmarks or non-obstructive metal artifacts;  Excluding patients with malignant tumours, fractures, post-surgery conditions or metallic implants that obstructed landmark identification;  Data were collected internally from the Department of Radiology at West China Hospital of Stomatology (September 2020 – October 2022) and externally from Tianfu Hospital and Jinjiang Outpatient Clinic (November 2022 – December 2023). |

**Table 1 (continued)**

| **Year, author** | **Quantity of CBCT/CT** | **Imaging Device; CBCT/CT parameters** | **Age (y)/dentition type; data source, presence and classification of malocclusion** |
| --- | --- | --- | --- |
| 2026, Baldini et al.^15^ | 350/0 | i–CAT (Imaging Sciences International): tube current: 5–48 mA, tube voltage: 80–120 kV, voxel size: 0.4 mm, 0.183 mm, 0.25 mm, 0.3 mm;  ProMax (Planmeca): tube current: 4–5 mA, tube voltage: 100–120 kV, voxel size: 0.2 mm, 0.4 mm;  WhiteFox Control (de Götzen S.r.l.): tube current: 10 mA, tube voltage: 105 kV, voxel size: 0.3 mm | 7–58, mean age=16±12/MD+PD;  Patient for orthodontic and maxillofacial treatment from September 2017 to July 2023 at the University of Milan’s Department of Biomedical Surgical and Dental Sciences; The dataset consisted of Caucasian subjects |

Note: NM, not mentioned; MD, mixed dentition; CT, computed tomography; CBCT, cone-beam computed tomography; PD, permanent dentition; DD, deciduous dentition; NewTom VGi CBCT, NewTom VGi CBCT machine (QR SRL Company, Verona, Italy); iCAT Next Generation CBCT, iCAT Next Generation CBCT unit (Imaging Sciences International, Hatfield, PA); FOV, field of view
