# Supplementary material for: Automatic identification of anatomical landmarks in three-dimensional computed tomography/cone-beam computed tomography: a scoping review
Source: Front Dent Med. 2026 May 29;7:1847046. doi: 10.3389/fdmed.2026.1847046 (PMC13260512; doi:10.3389/fdmed.2026.1847046)
Supplement: Supplementary file 2 [file Table2.docx]

**Table 2.** Number of anatomical landmarks and marking methods in the included literature

| **Year, author** | **Total number**  **of landmarks (hard tissue/soft tissue)** | **Annotation methodology and software** | **Annotator** |
| --- | --- | --- | --- |
| 2014, Shahidi et al.^26^ | 14 (14/0) | 3D-surface-rendered model+MPR | Three experts (two orthodontists and one maxillofacial radiologist) independently located landmarks twice, with a 6-week interval. Intraobserver ICC: 0.89; interobserver ICC: 0.87 |
| 2015, Gupta et al.^27^ | 20 (20/0) | Volume-rendered view+MPR | Three orthodontists; ICC >0.9 |
| 2016, Zhang et al.^10^ | 15 (15/0) | NM | An experienced CMF surgeon |
| 2016, Gupta et al.^28^ | 21 (21/0) | Volume rendered image+MPR; MIMICS (Materialise, Belgium) software | Three orthodontists; landmarks were plotted over a period of two weeks’ time. Inter-observer ICC >0.9 |

**Table 2 (continued)**

| **Year, author** | **Total number**  **of landmarks (hard tissue/soft tissue)** | **Annotation methodology and software** | **Annotator** |
| --- | --- | --- | --- |
| 2017, Codari et al.^40^ | 21 (21/0) | MPR; MATLAB (MathWorks, Natick, MA, USA) (guided user interface, GUI) | Three expert operators (at least 4 years of experience in morphological evaluation of the skull). The skull was automatically annotated in a double-blind process for three times to take intra- and inter-operator variability into account. The mean (standard deviation) inter-operator ICC was 0.98 (0.04) |
| 2017, Zhang et al.^33^ | 15 (15/0) | 3D rendered reconstruction model+MPR; MIMICS software | Two experienced CMF surgeons |
| 2018, Montúfar et al.^11^ | 18 (18/0) | 3D visualization+MPR; Matlab (MathWorks, Natick, Mass)+3DSlicer | Manual annotation was independently made twice by 2 observers. From 1 researcher, intraoperator data were obtained by placing 10 times |

**Table 2 (continued)**

| **Year, author** | **Total number**  **of landmarks (hard tissue/soft tissue)** | **Annotation methodology and software** | **Annotator** |
| --- | --- | --- | --- |
| 2018, Montúfar et al.^12^ | 18 (18/0) | 3D rendered reconstruction model+MPR; MATLAB+3DSlicer | Manual annotation was independently made twice by 2 observers with varying landmarking experience |
| 2018, Neelapu et al.^30^ | 20 (20/0) | 3D rendered reconstruction model+MPR; MIMICS software (Materialise, Leuven, Belgium); MATLAB (MathWorks, Inc.) | Three experienced orthodontists (two had a clinical and research experience of 8 years and another had of 5 years); ICC >0.98 |
| 2018, Jong et al.^29^ | 33 (33/0) | NM | NM |
| 2019, Lee et al.^13^ | 7 (7/0) | SimPlant software | Two experts who have been working on 3D cephalometry for more than ten years. ICC for intra-observer was 0.95 and for inter-observer was 0.92 |

**Table 2 (continued)**

| **Year, author** | **Total number**  **of landmarks (hard tissue/soft tissue)** | **Annotation methodology and software** | **Annotator** |
| --- | --- | --- | --- |
| 2019, O’Neil et al.^42^ | 22 (22/0) | NM | Two observers, one of whom (observer A, L.C.) has also annotated a large number of the training scans, the second of whom (observer B, E.S.) was independent of the training data |
| 2019, Torosdagli et al.^37^ | 9 (9/0) | 3D rendered reconstruction model+MPR; 3D Slicer software | Three expert interpreters (one from the NIH team, two from the UCF team); two experts (from the UCF team) repeated their manual annotations (after one month period of their initial annotations) for intra-observer evaluations, ICC=0.92 |
| 2020, Ma et al.^14^ | 13 (13/0) | 3D rendered reconstruction model+MPR; MIMICS software (Materialise NV, Version 21) | One of the authors |

**Table 2 (continued)**

| **Year, author** | **Total number**  **of landmarks (hard tissue/soft tissue)** | **Annotation methodology and software** | **Annotator** |
| --- | --- | --- | --- |
| 2020, Zhang et al.^34^ | 15 (15/0) | 3D rendered reconstruction model+MPR; MIMICS software (Materialise, Leuven, Belgium) | Two experienced CMF surgeons |

| 2021, Chen et al.^43^ | 18 (18/0) | 3D rendered reconstruction model; AnatomicAligner | Experienced CMF surgeons |
| --- | --- | --- | --- |

| 2022, Dot et al.^35^ | 33 (33/0) | NM | Landmarks were annotated, either once by operator 1 (a trained orthodontist with 5 years of clinical experience) or twice by operators 1, 2 (a trained orthodontist with 5 years of clinical experience), and 3 (a final year postgraduate maxillofacial surgeon) |
| --- | --- | --- | --- |
| 2022, Yun et al.^24^ | 90 (90/0) | NM | S.-H. Lee (an expert in 3D cephalometry with more than 20 years of experience) |

**Table 2 (continued)**

| **Year, author** | **Total number**  **of landmarks (hard tissue/soft tissue)** | **Annotation methodology and software** | **Annotator** |
| --- | --- | --- | --- |
| 2022, Ghowsi et al.^6^ | 53 (53/0) | 3D surface-rendered model+MPR; Checkpoint software (Stratovan Corporation, Davis, Calif) | Two judges independently performed landmark identification. Judge 1 was an experienced orthodontic resident with at least 2 years of experience in landmark identification on 3D imaging. Judge 2 was an oral and maxillofacial radiologist who has been considered an expert in 3D imaging for more than 20 years |
| 2022, Chen et al.^25^ | 17 (17/0) | 3D rendered reconstruction model+MPR | NM |
| 2022, Lang et al.^23^ | 105 (105/0) | AnatomicAligner software | Two experienced CMF surgeons |
| 2023, Gillot et al.^31^ | 32 (32/0) | ITK-SNAP (version 3.8)+3D Slicer (version 4.11) | Two clinician experts |

**Table 2 (continued)**

| **Year, author** | **Total number**  **of landmarks (hard tissue/soft tissue)** | **Annotation methodology and software** | **Annotator** |
| --- | --- | --- | --- |
| 2023, Xu et al.^22^ | 32 (32/0) | 3D rendered reconstruction model; MIMICS software (version 20.0; Materialise, Leuven, Belgium) | All 3 operators had more than 5 years of experience in craniomaxillofacial surgery |
| 2023, Xu et al.^21^ | 27 (27/0) | 3D rendered reconstruction model; MIMICS software (version 20.0; Materialise) | All 3 operators had more than 5 years of experience in craniomaxillofacial surgery |
| 2023, Tao et al.^16^ | 77 (64/13) | 3D rendered reconstruction model; MIMICS software (Materialise, Belgium) | Manually digitized: 2 junior CMF surgeons;  modified: a senior CMF surgeon |

**Table 2 (continued)**

| **Year, author** | **Total number**  **of landmarks (hard tissue/soft tissue)** | **Annotation methodology and software** | **Annotator** |
| --- | --- | --- | --- |
| 2023, Blum et al.^39^ | 35 (35/0) | MPR | All four experts were experienced orthodontists. All CBCTs were analyzed by three of the four experienced experts in a random order. To investigate the reproducibility of the manual landmark detection and the intraindividual variation of the individual experts, CBCT datasets were unknowingly analyzed twice |
| 2024, Wang et al.^32^ | 27 (27/0) | 3D rendered reconstruction model+ MyDentViewer software (version 1.0; Meyer, Hefei, Anhui) | NM |

**Table 2 (continued)**

| **Year, author** | **Total number**  **of landmarks (hard tissue/soft tissue)** | **Annotation methodology and software** | **Annotator** |
| --- | --- | --- | --- |
| 2024, Sahlsten et al.^38^ | 46 (46/0) | Finnish cohort: Romexis 4.6.2 software (Planmeca Oy, Helsinki, Finland),  Thai cohort: OnDemand 3D software (Cybermed Co., Seoul, Korea) | Finnish cohort: a specialist in dental and maxillofacial radiology, a resident in orthodontics with several years of experience in surgical planning using cephalometric landmarks and a resident in dental and maxillofacial radiology with several years of experience in 2D and 3D cephalometry;  Thai cohort: two specialists in dental and maxillofacial radiology with several years of experience in 2D cephalometry |
| 2024, Tao et al.^36^ | 77 (64/13) | NM | Manually digitized: two junior CMF surgeons, subsequent reviewed: a senior CMF surgeon |
| 2024, Park et al.^44^ | 65 (55/10) | NM | 2 biomedical experts, under the supervision of a clinician |

**Table 2 (continued)**

| **Year, author** | **Total number**  **of landmarks (hard tissue/soft tissue)** | **Annotation methodology and software** | **Annotator** |
| --- | --- | --- | --- |
| 2025, Tanikawa et al.^41^ | 64 (64/0) | HBM Rugle (Medical Engineering Inc., Kyoto, Japan) | An experienced orthodontist (YU) manually identified landmarks, another experienced orthodontist (CT; university faculty) checked |
| 2025, Zhu et al.^18^ | 53 (44/9) | NM | Two senior orthodontists, one with 9 years and the other with 14 years of experience, subsequently verified by a chief orthodontist with 31 years of experience |
| 2025, Gao et al.^45^ | 20–25 (60/0) | A custom annotation tool | Three experienced orthodontists (≥10 year of experience) used a custom annotation tool. Each landmark was independently marked by two specialists, with discrepancies exceeding 1.0 mm resolved by consensus discussion with the third specialist. |

**Table 2 (continued)**

| **Year, author** | **Total number**  **of landmarks (hard tissue/soft tissue)** | **Annotation methodology and software** | **Annotator** |
| --- | --- | --- | --- |
| 2025, Deitermann et al.^19^ | 9 (9/0) | Mimics 14 (Materialise Inc., Leuven, Belgium) | NM |
| 2025, Jiang et al.^20^ | 43 (32/11) | Dolphin 3D software (Dolphin Imaging and Management Systems, Chatsworth, CA, USA); Checkpoint software (ITK, Davis, Calif, USA) | Judge 1, an orthodontist and Judge 2, an oral and maxillofacial surgeon (both considered experts with >10 years of experience in landmark identification in 2D and 3D imaging) independently performed manual landmark identification |

**Table 2 (continued)**

| **Year, author** | **Total number**  **of landmarks (hard tissue/soft tissue)** | **Annotation methodology and software** | **Annotator** |
| --- | --- | --- | --- |
| 2025, Liu et al.^17^ | 55 (46/9) | 3D rendered reconstruction model+Mimics 16.0 (Materialize Interactive Medical Image Control System, Belgium). | SCT images were independently annotated by a senior oral and maxillofacial surgeon (B.Y.L., 9 years of experience); CBCT images were annotated by a senior orthodontist (Ch.L., 14 years of experience). All landmarks underwent rigorous quality control by a senior radiologist (W.T., 31 years of experience) |
| 2026, Baldini et al.^15^ | 16 (16/0) | 3DSlicer (Brigham and Women’s Hospital, version 5.2.2) | An expert orthodontist (MS) with >5 years of experience |

Note: NM, not mentioned; MPR, multiplanar reformation
