# Supplementary material for: Automatic identification of anatomical landmarks in three-dimensional computed tomography/cone-beam computed tomography: a scoping review
Source: Front Dent Med. 2026 May 29;7:1847046. doi: 10.3389/fdmed.2026.1847046 (PMC13260512; doi:10.3389/fdmed.2026.1847046)
Supplement: Supplementary file 3 [file Table3.docx]

**Table 3.** Automatic landmarking methods and accuracy of the included studies

| **Year, author** | **General method** | **Algorithms used** | **MRE±SD (mm)** | | **SDR < 2 mm** |
| --- | --- | --- | --- | --- | --- |
| 2014, Shahidi et al.^26^ | Atlas-based | A combination of feature-based (principal axes registration) and voxel similarity-based methods for image registration | 3.40 mm | | NM |
| 2015, Gupta et al.^27^ | Knowledge-based | Knowledge-Based Automatic Landmark Detection Algorithm | 2.01±1.23 mm | | 64.67% |
| 2016, Zhang et al.^10^ | Learning-based | Random forest, Segmentation-Guided Partially-Joint Regression Forest Model (S-PRF) | 1.44 mm | | NM |
| 2016, Gupta et al.^28^ | Knowledge-based | Knowledge-Based Automatic Landmark Detection Algorithm | NM | | NM |
| 2017, Codari et al.^40^ | Atlas-based | Intensity-Based Image Registration | NM | | NM |
| 2017, Zhang et al.^33^ | Learning-based | Deep Leaning, Context-Guided Fully Convolutional Networks | 1.10±0.71 mm | NM | |

**Table 3 (continued)**

| **Year, author** | **General method** | **Algorithms used** | **MRE±SD (mm)** | **SDR < 2 mm** |
| --- | --- | --- | --- | --- |
| 2018, Montúfar et al.^11^ | Learning-based | Active Shape Model (ASM) | 3.65±1.43 mm | NM |
| 2018, Montúfar et al.^12^ | Learning-based method, Knowledge-based method | Hybrid Active Shape Model with Knowledge-based Landmark Localization | 2.51±1.61 mm | NM |
| 2018, Neelapu et al.^30^ | Knowledge-based | Automatic Detection of Landmarks Based on Anatomical Boundary Definitions and Symmetry Features of the Skull | 1.88±1.10 mm | 64.16% |
| 2018, Jong et al.^29^ | Learning-based | Gabor wavelets and ensemble learning | NM | NM |
| 2019, Lee et al.^13^ | Learning-based | Deep Learning, Automatic 3D Cephalometric Annotation System Using Shadowed 2D Image-Based Machine Learning | 1.5 mm | 90.50% |

**Table 3 (continued)**

| **Year, author** | **General method** | **Algorithms used** | **MRE±SD (mm)** | **SDR < 2 mm** |
| --- | --- | --- | --- | --- |
| 2019, O’Neil et al.^42^ | Learning-based | Deep Learning, Atlas Location Autocontext for Anatomical Landmark Detection | NM | NM |
| 2019, Torosdagli et al.^37^ | Learning-based | Deep Learning, Deep Geodesic Learning | NM | NM |
| 2020, Ma et al.^14^ | Learning-based | Deep Learning, Patch-based Deep Neural Network | 5.79±0.98 mm | NM |
| 2020, Zhang et al.^34^ | Learning-based | Deep Learning, Context-guided Fully Convolutional Network (FCN) | 1.10±0.71 mm | NM |
| 2021, Chen et al.^43^ | Learning-based | 3D faster R-CNN+MS-UNet | 0.89±0.64 mm | NM |
| 2022, Dot et al.^35^ | Learning-based | Deep Learning, SpatialConfiguration-Net (SCN) | 1.0±1.3 mm | 90.40% |
| 2022, Yun et al.^24^ | Learning-based | Deep Learning, Semi-supervised Learning Approach | 2.88 mm | NM |
| 2022, Ghowsi et al.^6^ | Learning-based | Deep Learning, Automated Landmark Identification (ALI) | 3.19±2.60 mm | 35% |

**Table 3 (continued)**

| **Year, author** | **General method** | **Algorithms used** | **MRE±SD (mm)** | **SDR < 2 mm** |
| --- | --- | --- | --- | --- |
| 2022, Chen et al.^25^ | Learning-based | Deep Learning, Structure-Aware Long Short-Term Memory Network (SA-LSTM) | 1.64±1.13 mm | 74.28% |
| 2022, Lang et al.^23^ | Learning-based | Deep Learning, 3D Mask R-CNN and Local Dependency Learning | 1.38±0.95 mm | NM |
| 2023, Gillot et al.^31^ | Learning-based | Deep Learning, Automatic Landmark Identification in Cone-Beam Computed Tomography | 1.54±0.87 mm | NM |
| 2023, Xu et al.^22^ | Learning-based | Deep Learning, Graph Convolutional Network (GCN) | 1.46±1.31 mm | 90% |
| 2023, Xu et al.^21^ | Learning-based | Deep Learning, Graph Convolutional Network (GCN) | 1.33 mm | NM |
| 2023, Tao et al.^16^ | Learning-based | Deep learning, 3D UX-Net | 1.81±0.89 mm | 76.62% |

**Table 3 (continued)**

| **Year, author** | **General method** | **Algorithms used** | **MRE±SD (mm)** | **SDR < 2 mm** |
| --- | --- | --- | --- | --- |
| 2023, Blum et al.^39^ | Learning-based | Deep learning, U-Net and CNN | 2.73 mm | NM |
| 2024, Wang et al.^32^ | Learning-based | Deep Learning, PointRend, PoseNet, template mapping technique | 1.04±0.28 mm | NM |
| 2024, Sahlsten et al.^38^ | Learning-based | Deep Learning, stacked hourglass network | NM | 63% |
| 2024, Tao et al.^36^ | Learning-based | Deep Learning, a semi-supervised learning (SSL) model, named CephaloMatch | 1.60±0.87 mm | NM |
| 2024, Park et al.^44^ | Learning-based | Deep learning | NM | NM |

**Table 3 (continued)**

| **Year, author** | | **General method** | | **Algorithms used** | | **MRE±SD (mm)** | | **SDR < 2 mm** |
| --- | --- | --- | --- | --- | --- | --- | --- | --- |
| 2025, Tanikawa et al.^41^ | | Learning-based | | Deep Learning, Learning-Based Local-To-Global Landmark Annotation, 3D Faster R-CNN, Multiple Stage Deep Reinforcement Learning and PointNet++ | | 2.15±1.38 mm | | NM |
| 2025, Zhu et al.^18^ | | Learning-based | | Deep Learning, multiscale global information extraction module (MSGIEM), landmark attention module (LAM), 3-D U-Net | | CBCT: 1.20±0.32 mm;  CT: 1.40±0.47 mm | | CBCT: 87.17%;  CT: 81.60% |
| 2025, Gao et al.^45^ | | Learning-based | | DeepFuse (a novel multi-modal deep learning framework) | | 1.21±0.58 mm (multi-modal) | | 92.4% (multi-modal) |
| 2025, Deitermann et al.^19^ | Learning-based | | Two-stage coarse-to-fine pipeline  Dual-input 3D U-Net | | NM | | NM | |

**Table 3 (continued)**

| **Year, author** | **General method** | **Algorithms used** | **MRE±SD (mm)** | **SDR < 2 mm** |
| --- | --- | --- | --- | --- |
| 2025, Jiang et al.^20^ | Learning-based | Deep Learning, U-Net, Efficient Global Attention (EGA) module | 1.76±1.13 mm | 60.16% |
| 2025, Liu et al.^17^ | Learning-based | Deep Learning, Optimized lightweight 3D U-Net | CBCT: 1.012±0.530 mm;  spiral CT: 1.22±0.72 mm | CBCT: 93.3%;  spiral CT: 90.5% |
| 2026, Baldini et al.^15^ | Learning-based | Deep learning, 3D fully CNN, a light-weight deep learning (DL) model, V-net | 1.77±0.97 mm | NM |

Note: NM, not mentioned; MRE, mean radial error; SD, standard deviation; SDR, successful detection rate
